# Comparative analysis of EpCAM high-expressing and low-expressing circulating tumour cells with regard to their clonal relationship and clinical value

**DOI:** 10.1038/s41416-023-02179-0

**Published:** 2023-02-23

**Authors:** André Franken, Annika Kraemer, Alicia Sicking, Meike Watolla, Mahdi Rivandi, Liwen Yang, Jens Warfsmann, Bernhard M. Polzer, Thomas W. P. Friedl, Franziska Meier-Stiegen, Nikolas H. Stoecklein, Davut Dayan, Sabine Riethdorf, Volkmar Mueller, Klaus Pantel, André Koch, Andreas D. Hartkopf, Natalia Krawczyk, Eugen Ruckhaeberle, Dieter Niederacher, Tanja Fehm, Hans Neubauer

**Affiliations:** 1 Department of Obstetrics and Gynecology, University Hospital and Medical Faculty of the Heinrich-Heine University Duesseldorf, Duesseldorf, Germany; 2 Division “Personalized Tumor Therapy”, Fraunhofer Institute for Toxicology and Experimental Medicine, Regensburg, Germany; 3 Department of Gynecology and Obstetrics, University Hospital Ulm, Ulm, Germany; 4 General, Visceral and Pediatric Surgery, University Hospital and Medical Faculty of the Heinrich Heine University Duesseldorf, Duesseldorf, Germany; 5 Department of Tumor Biology, University Hospital Hamburg-Eppendorf, Hamburg, Germany; 6 Department of Gynecology, University Medical Centre Hamburg-Eppendorf, Hamburg, Germany; 7 Department of Obstetrics and Gynecology, University of Tuebingen, Tuebingen, Germany

**Keywords:** Predictive markers, Breast cancer

## Abstract

**Background:**

Circulating tumour cells (CTCs) are mainly enriched based on the epithelial cell adhesion molecule (EpCAM). Although it was shown that an EpCAM low-expressing CTC fraction is not captured by such approaches, knowledge about its prognostic and predictive relevance and its relation to EpCAM-positive CTCs is lacking.

**Methods:**

We developed an immunomagnetic assay to enrich CTCs from metastatic breast cancer patients EpCAM independently using antibodies against Trop-2 and CD-49f and characterised their EpCAM expression. DNA of single EpCAM high expressing and low expressing CTCs was analyzed regarding chromosomal aberrations and predictive mutations. Additionally, we compared CTC-enrichment on the CellSearch system using this antibody mix and the EpCAM based enrichment.

**Results:**

Both antibodies acted synergistically in capturing CTCs. Patients with EpCAM high-expressing CTCs had a worse overall and progression-free survival. EpCAM high- and low-expressing CTCs presented similar chromosomal aberrations and mutations indicating a close evolutionary relationship. A sequential enrichment of CTCs from the EpCAM-depleted fraction yielded a population of CTCs not captured EpCAM dependently but harbouring predictive information.

**Conclusions:**

Our data indicate that EpCAM low-expressing CTCs could be used as a valuable tumour surrogate material—although they may be prognostically less relevant than EpCAM high-expressing CTCs—and have particular benefit if no CTCs are detected using EpCAM-dependent technologies.

## Background

Circulating tumour cells (CTCs) are shed into the blood by tumour tissue and are commonly considered as precursors of distant metastatic spread. They offer the opportunity to act as an analyte in a blood-based liquid biopsy [1]. Elevated CTC counts correlate with shortened progression-free survival (PFS) and overall survival (OS) in metastatic and non-metastatic breast cancer and other metastatic cancers [2, 3]. The predictive utility of CTCs is currently being investigated: Recent studies such as the STIC-CTC trial or the DETECT-III trial indicate that both their counts and phenotypes such as HER2/neu positivity can be used for drawing decisions to treat breast cancer [4, 5]. Moreover, CTCs—as they are intact and living cells—could serve as a biomarker suitable for multiparametric analyses [6].

The enrichment of CTCs is highly challenging due to their rareness of only 1 CTC per millions of white blood cells (WBCs)—even in the metastatic situation. It is mainly based on immunomagnetic technologies relying on antibodies against the epithelial cell adhesion molecule (EpCAM) such as in the CellSearch system, which is the only FDA-approved technology to enrich CTCs from blood of breast, prostate, colorectal, or lung cancer patients [7]. However, in breast cancer and other cancers a subpopulation of CTCs with low or absent EpCAM expression exists, which may be a consequence of processes leading to a phenotypic plasticity such as the epithelial-to-mesenchymal transition [8]—a highly conserved mechanism which is associated with cell migration, invasion and dissemination in the development of cancer metastasis [9]. Consequently, the subpopulation of EpCAM low-expressing CTCs is only poorly captured by EpCAM-dependent approaches [10]. Some studies even indicate that the EpCAM-negative CTCs exceed the EpCAM positive in quantity in e.g., metastatic lung cancer [11].

To circumvent such limitations several EpCAM-independent technologies are available. These technologies can be based on a negative depletion of CD45-positive blood cells of hematopoietic origin [12] or on positive CTC enrichment exploiting physical properties such as their larger size compared to WBCs and their less deformable cell morphology in filters or microfluidic chips [13, 14]. Furthermore, CTCs can be captured by immunomagnetic technologies addressing other surface proteins than EpCAM [15].

Despite many approaches to enrich CTCs EpCAM independently, knowledge about their prognostic and predictive relevance and their relation to EpCAM-positive CTCs is still lacking and the results of studies in this field are contradictory. Whereas some studies indicate the prognostic relevance of EpCAM-negative CTCs [16] others could not show any association of EpCAM-negative CTCs with patients’ outcomes [17].

To shed light on the clonal relationship of EpCAM low- and high-expressing CTCs as well as on the prognostic and predictive relevance of EpCAM low-expressing CTCs we developed an immunomagnetic assay that co-enriches EpCAM high-expressing and EpCAM low-expressing CTC populations independently of their physical properties with the same method for a direct comparison. This was realised by targeting Trop-2 as a surface protein predominantly expressed in EpCAM high-expressing CTCs and CD-49f as a surface protein particularly present on EpCAM low-expressing CTCs. Based on that we were able to investigate the CTCs’ clonal relationship, the presence of predictive information such as mutations, as well as their morphology. The results indicate that EpCAM low-expressing CTCs may be a valuable biomarker of a blood-based liquid biopsy.

## Results

### Selection of markers for an EpCAM-independent positive enrichment of CTCs

Schneck et al. showed that antibodies targeting several surface proteins were suitable to enrich CTCs from the EpCAM-depleted fraction of the CellSearch system [15]. Based on these data we aimed to combine one antibody targeting surface proteins on epithelial-like CTCs representing an EpCAM high- expressing phenotype and one antibody targeting surface proteins on mesenchymal-like CTCs representing an EpCAM low-expressing phenotype.

For target protein selection, the expression of the respective targets was correlated with the *EPCAM* expression based on two publicly available datasets from EpCAM independently enriched breast cancer CTCs [18, 19]. Although antibodies targeting EpCAM were included in CTC detection, detection was not based exclusively on EpCAM allowing the analysis of *EPCAM*-negative or low-expressing CTCs. Indeed, on RNA level, *EPCAM* positivity was observed for only 55.6% and 36.4% of the cells, respectively.

**Fig. 1:**
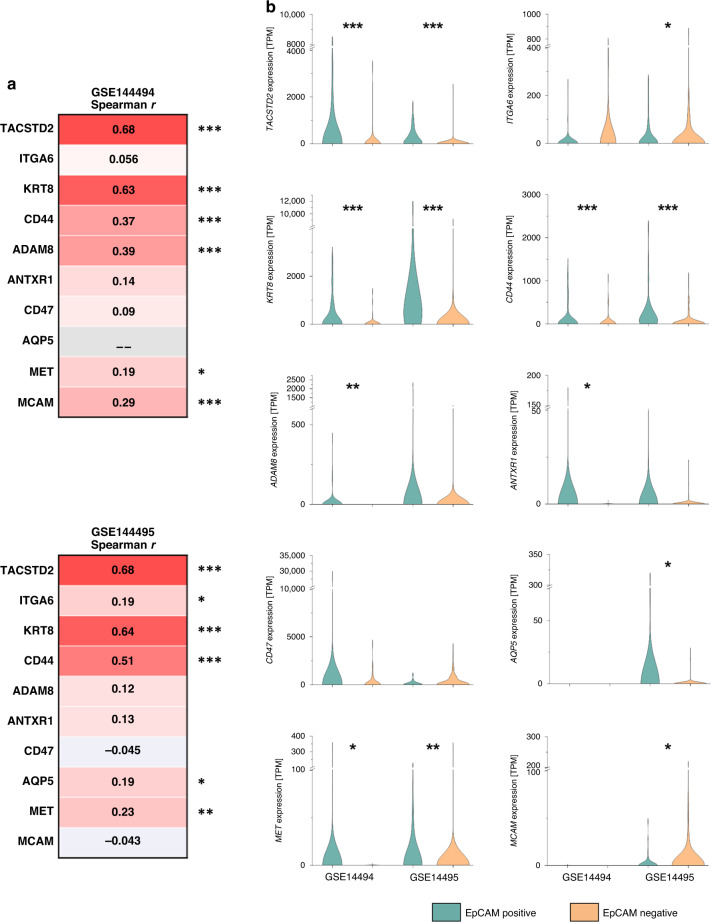
Expression of targets for CTC enrichment. Gene expression data from 135 CTCs and 195 CTCs, respectively, were obtained from gene expression omnibus (national centre for biotechnology information; GSE144494 and GSE144495). **a** Correlation coefficients for the correlation between the expression of EpCAM and the respective CTC enrichment marker in CTCs were calculated by non-parametric Spearman correlation. The *P* values were adjusted to multiple testing by Benjamini–Hochberg correction (* indicates a *P* value <0.05, ** indicates a *P* value <0.01, *** indicates a *P* value <0.001). **b** Comparison of the expression of the respective CTC enrichment marker in EpCAM-positive and -negative CTCs. Significance levels were determined by the two-sided Mann–Whitney test and were adjusted to multiple testing by Benjamini–Hochberg correction.

A high correlation of *EPCAM* expression with *TACSTD2* (0.68 and 0.68), *KRT8* (0.63 and 0.64), and CD44 (0.37 and 0.51) expression was observed. Among others, we found a particularly low correlation of the expression of *EPCAM* with the expression of *ITGA6* (0.056 and 0.19) (Fig. 1a).

Moreover, we observed *TACSTD2*, *KRT8*, *CD44* and *MET* to be significantly upregulated in the *EPCAM*-positive CTCs in both datasets suggesting that antibodies targeting those markers may be particularly capturing EpCAM-positive CTCs. *ADAM8*, *ANTXR1* and *MCAM* were upregulated in the *EPCAM*-positive CTCs in one dataset and not significantly differently expressed in the other. The only marker significantly (*P* = 0.0417) higher expressed in the EPCAM-negative CTCs in one dataset and showing a strong tendency towards an upregulation in the EPCAM-negative fraction in the other dataset was *ITGA6*, indicating the ability of its protein CD-49f to serve as a target for the enrichment of EpCAM-negative or low-expressing CTCs (Fig. 1b).

Based on these results and considering that Schneck et al. observed particularly high CTC yields applying antibodies targeting Trop-2, the protein coded by the gene *TACSTD2*, and CD-49f, we selected these proteins to serve as targets to enrich both subgroups of EpCAM high- and low-expressing CTCs.

### The combined use of Trop-2 and CD-49f-directed antibodies enriches CTCs synergistically

Next, we established a workflow to enrich CTCs immunomagnetically applying antibodies targeting Trop-2 and CD-49f. Therefore, the breast cancer cell lines SK-BR-3, T-47D and MCF7 were selected as Trop-2 high- and CD-49f low-expressing controls and the cell line MDA-MB-231 was used as a model for CD-49f high- and Trop-2 low-expressing tumour cells, shown by flow cytometry. Furthermore, the antibody targeting Trop-2 did not bind to WBCs. The antibody targeting CD-49f showed hardly any signal on WBCs (Supplemental Fig. 1). The slight binding observed results from a weak expression of the protein on some eosinophils [20].

**Fig. 2:**
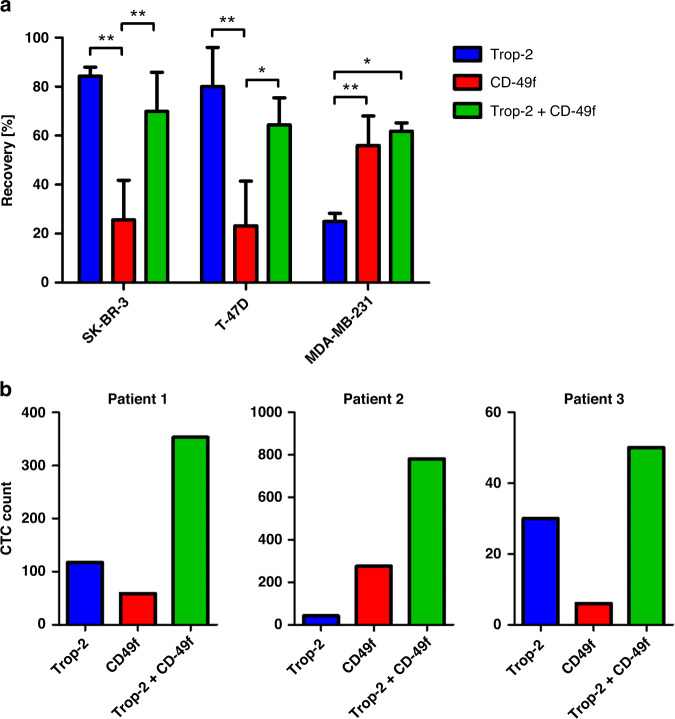
CTC enrichment using antibodies targeting Trop-2 and CD-49f. **a** Prestained cells from SK-BR-3, T-47D and MDA-MB-231 cell lines were spiked into healthy donors’ blood samples and Trop-2 and/or CD-49f-based recovery rates on the Isoflux system were determined. Error bars indicate standard deviation. The *P* values were determined by one-way ANOVA and posthoc Bonferroni test (* indicates a *P* value <0.05, ** indicates a *P* value <0.01, *** indicates a *P* value <0.001). The data were generated in three independent replicates. **b** CTCs were enriched from 5 ml of blood each either Trop-2-based, CD-49f-based, or CD-49f- and Trop-2 based.

In contrast to Schneck et al., we did not bind the antibodies to beads but first incubated the antibodies with the cells and then added the beads in an indirect manner. Applying each antibody alone to healthy donors’ blood samples with spiked prestained cells from cell lines, we achieved recovery rates of 84.3% and 80.1% for SK-BR-3 and T-47D cells captured with the antibody targeting Trop-2 and 55.9% for MDA-MB-231 cells captured with the antibody targeting CD-49f. As expected, targeting CD-49f on SK-BR-3 and T-47D cells or Trop-2 on MDA-MB-231 cells led to significantly lower yields. Combining both antibodies did not influence the recovery of cells from all three cell lines significantly (*P* > 0.05) (Fig. 2a). Binding of the isotype control or applying empty beads did not result in tumour cell enrichment.

Afterwards, unstained cells from SK-BR-3 and T-47D cell lines were spiked into healthy donors’ blood to mimic a real situation. Tumour cells were recovered, stained for cytokeratins, EpCAM, CD45, and with a Hoechst dye, and detached from beads leading to recovery rates of 45.2% for SK-BR-3 and 44.0% for T-47D cells (Supplemental Fig. 2). As a control, samples from five age-matched female healthy donors were analysed and no cytokeratin-positive/CD45-negative cells were detected.

Next, the workflow was applied to blood samples from three metastatic breast cancer patients. For each patient, CTCs were enriched with each antibody alone and with both antibodies combined. For CTCs, the simultaneous usage of both antibodies achieved a synergistic effect over the single antibodies in each patient: The CTC count increased considerably when both antibodies were used simultaneously (Fig. 2b). Of note, CTCs captured with the antibody targeting Trop-2 showed a higher EpCAM expression than CTCs captured with the antibody binding to CD-49f indicating that the former binds to EpCAM high-expressing CTCs and the latter targets EpCAM low-expressing CTCs indeed (Supplemental Fig. 3).

### EpCAM high and EpCAM low-expressing CTCs represent similar tumour cell clones

**Fig. 3:**
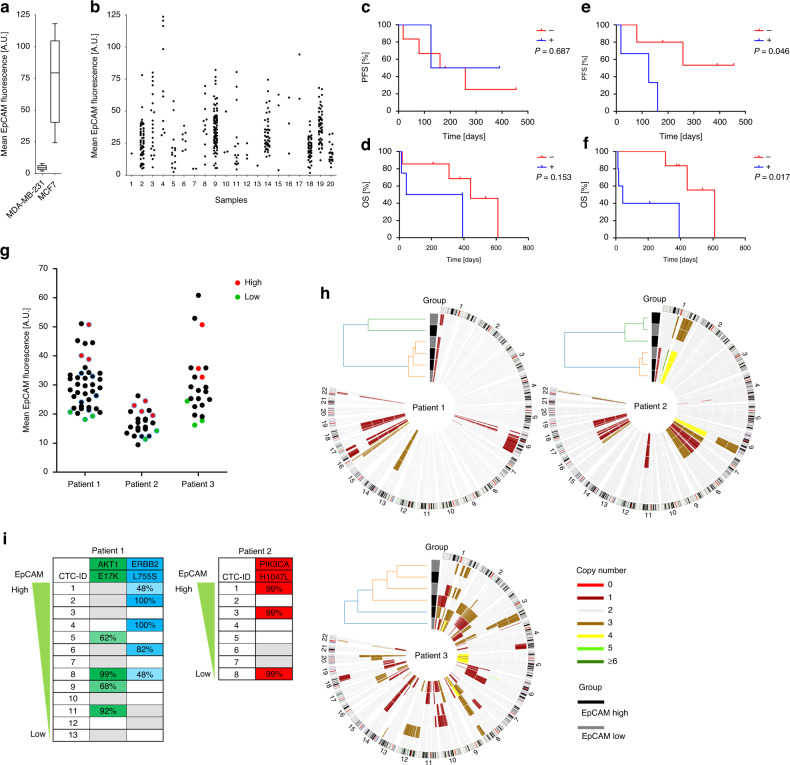
Comparative analysis of EpCAM high- and low-expressing CTCs. **a** Cells from MCF7 and MDA-MB-231 cell lines were spiked into healthy donors’ blood samples, enriched based on Trop-2 and CD-49f and stained on glass slides. Anti-EpCAM mean fluorescence intensity (MFI) was determined as reference. **b** CTCs were enriched Trop-2/CD-49f based, stained on glass slides, and the anti-EpCAM MFI was determined. Kaplan–Meier plots of progression-free survival (PFS) (**c**) and overall survival (OS) (**d**) of patients positive or negative for CTCs with an anti-EpCAM MFI below 12.5. Kaplan–Meier plots of progression-free (**e**) and overall survival (**f**) of patients positive or negative for CTCs with an anti-EpCAM MFI above 75. The *P* values were determined by the log-rank test. **g** CTCs from three independent patients were stained in suspension and isolated in order to analyse chromosomal aberrations and mutations. Upon whole-genome amplification (WGA) cells with high EpCAM expression (red) and low EpCAM expression (green) were selected for low-pass sequencing based on the WGA products’ quality. Further cells were analysed by mutation analysis (blue circled). **h** Chromosomal aberrations of intra-patient EpCAM high- and low-expressing CTCs from three patients were detected by low-pass sequencing. Dendrograms are based on hierarchical clustering. **i** Mutations in intra-patient EpCAM high- and low-expressing CTCs were detected by targeted next-generation sequencing. Values indicate variant allele frequency. A cut-off of 12.5% was applied. Grey boxes indicate that the respective region was not covered. No mutations were found for patient 3. A.U. artificial unit.

Next, the established workflow was applied to blood samples from metastatic breast cancer patients (patients’ characteristics are listed in Supplemental Table 1) and CTCs were stained for EpCAM. Cells from MDA-MB-231 and MCF7 cell lines that underwent capturing with our workflow were used as controls (Fig. 3a). Within the CTCs from 19 CTC-positive patients’ samples, we observed a wide spectrum of EpCAM low-, intermediate-, and high-expressing CTCs without distinct cutoffs to dichotomise the cells into high- and low-expressing CTCs. Instead, EpCAM high- and low-expressing CTCs appeared as two extremes of an expression continuum. Besides, the distribution of EpCAM high- and low-expressing CTCs differed considerably between the patients. In some patients such as patients 7 and 13 particularly CTCs with low EpCAM signals were found. In other patients such as patient 4 and 17 predominantly EpCAM high-expressing CTCs were detected. Further, some patients, e.g., patient 3, 9 and 11, presented CTCs of high and low EpCAM expression (Fig. 3b and Supplemental Fig. 4).

Afterwards, the patients’ outcome according to the presence of EpCAM high and low-expressing CTCs was investigated. We could not detect a significant effect of the presence of EpCAM low-expressing CTCs, characterised by a mean anti-EpCAM fluorescence intensity lower than 12.5, on the progression-free survival (PFS) or overall survival (OS) (Fig. 3c, d). To determine whether the presence of EpCAM high-expressing CTCs affected the patients’ outcome, different cutoffs of the mean anti-EpCAM fluorescence intensity were applied (Supplemental Fig. 5). A significant effect on the PFS and the OS was observed with a cut-off of 75. Patients presenting CTCs with EpCAM signal intensities exceeding this cut-off had a significantly reduced PFS and OS (Fig. 3e, f). Of note, we did not observe any significant association of the presence of EpCAM high or EpCAM low-expressing CTCs with patients’ clinical characteristics (Supplemental Table 2).

To compare the clonal relation of EpCAM high-expressing and low-expressing CTCs, cells representing each phenotype were isolated from three patients (patients’ characteristics are listed in Supplemental Table 3) and their chromosomal gains or losses were examined by low-pass sequencing. (Fig. 3g, h). Interestingly, in two EpCAM high-expressing CTCs no or almost no chromosomal aberrations were detected. Nevertheless, immunofluorescence signals clearly fulfilled the standard criteria for identifying a cell as a CTC (Supplemental Fig. 6). Upon applying hierarchical clustering, we did not observe a separation of EpCAM high and EpCAM low-expressing CTCs pointing towards their close phylogenetic relationship. Moreover, an analysis of hotspot mutations in the genes *PIK3CA*, *ESR1*, *AKT1* and *ERBB2* frequently present in breast cancer was performed. In the CTCs of patient 1, the mutations *AKT1* E17K and *ERBB2* L755S were detected. CTCs from patient 2 harboured the *PIK3CA* H1047L mutation. In both patients, the respective mutations were found in only a subgroup of the CTCs. However, we did not observe any association of EpCAM staining intensity and the presence of the mutations (Fig. 3i). In the CTCs from patient 3 no mutations were detected in the respective genes.

Consequently, our results suggest, that the EpCAM high-expressing and the EpCAM low-expressing CTC fractions originated from similar tumour cell clones.

### EpCAM dependently and EpCAM independently enriched CTCs represent a tumour surrogate of similar relevance

In some patients (namely patient 1, 7 and 13 from Fig. 3b), predominantly EpCAM low-expressing cells were observed which would very likely not be found with an EpCAM-dependent enrichment technology. To examine whether the established Trop-2 and CD-49f-based enrichment yields CTCs that cannot be found with an EpCAM-dependent technology, we aimed to compare both strategies on matched samples. As an EpCAM-dependent technology, the CellSearch system was used. To minimise workflow-dependent differences we established and optimised the Trop-2 and CD-49f-based enrichment on the CellSearch system, resulting in recovery rates of 61.15% for SK-BR-3 cells and 62.57% for MDA-MB-231 cells. The recovery rates were consistent independently of the number of spiked cells (Supplemental Fig. 7). As a control, samples from five age-matched female healthy donors were analysed and no CK-positive/CD45-negative cells were detected.

**Fig. 4:**
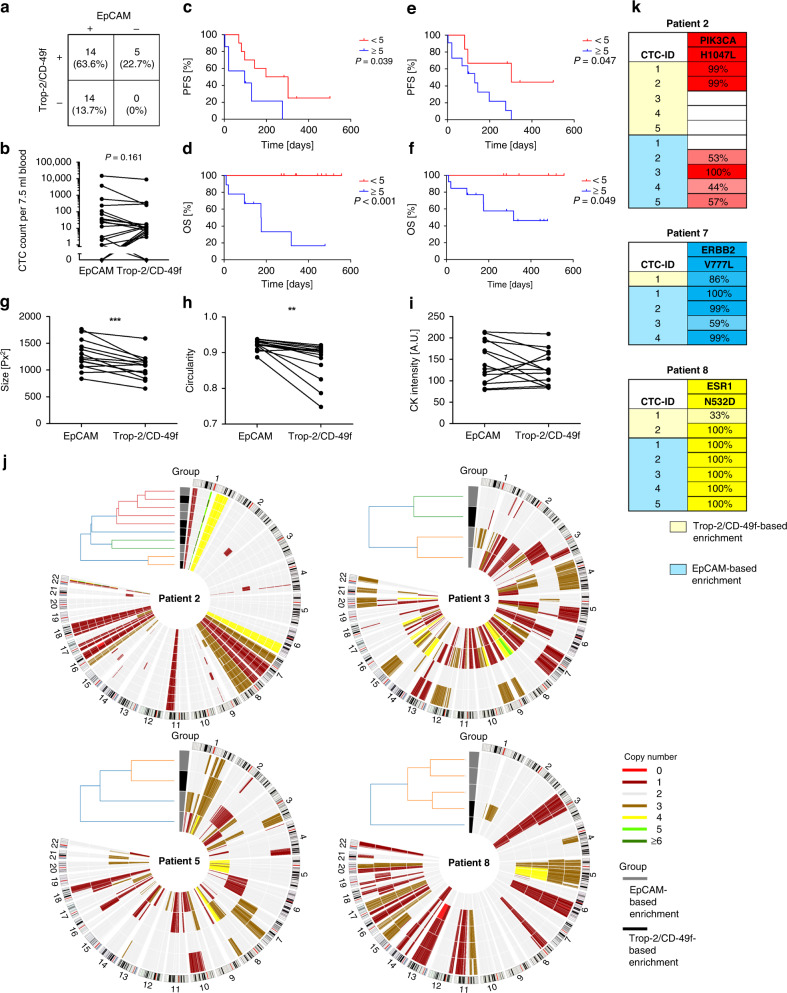
Comparative analysis of EpCAM dependently and independently enriched CTCs. **a** CTC positivity rates of EpCAM-based and Trop-2/CD-49f-based enrichment of 22 metastatic breast cancer patients. **b** CTC numbers of EpCAM-based and Trop-2/CD-49f-based enrichment. CTC numbers per 7.5 ml blood are shown. CTC numbers for the EpCAM-independent enrichment were extrapolated to 7.5 ml of blood. The *P* value was determined by paired two-sided *t* test. Kaplan–Meier plots of progression-free survival (PFS) (**c**) and overall survival (OS) (**d**) of patients with ≥5 CTCs versus patients with <5 CTCs per 7.5 ml blood based on the EpCAM-based enrichment. Kaplan–Meier plots of progression-free (**e**) and overall survival (**f**) of patients with ≥5 CTCs versus patients with <5 CTCs extrapolated to 7.5 ml blood based on the Trop-2/CD-49f-based enrichment. The *P* values were determined by the log-rank test. The CTCs’ morphology was compared with regard to their size (**g**), their circularity (**h**) and their anti-cytokeratin (CK) fluorescent intensity (**i**) (** indicates a *P* value <0.01, *** indicates a *P* value <0.001). Values show mean of each parameter for each patient. Comparisons of each patient’s CTCs is shown in Supplemental Fig. 6. The *P* values were determined by paired two-sided *t* test. **j** Chromosomal aberrations of EpCAM and Trop-2/CD-49f dependently enriched CTCs from four patients were detected by low-pass sequencing. Dendrograms are based on hierarchical clustering. **k** Mutations detected by targeted next-generation sequencing on DNA from EpCAM dependently (blue) and independently enriched CTCs (yellow). Values indicate the variant allele frequency. A cut-off of 12.5% was applied. The mutation data of all patients are shown in Supplemental Fig. 8.

Next, samples from 22 metastatic breast cancer patients were analysed (patients’ characteristics are listed in Supplemental Table 4). In 63.6% of the samples (14 patients), CTCs were detected with both enrichment strategies. In 13.7% (3 patients), CTCs were only detected in the EpCAM enriched fraction and in 22.7% (5 patients) we only found CTCs if antibodies against Trop-2 and CD-49f were applied. Overall, combining both enrichment strategies resulted in a CTC positivity of 100% (Fig. 4a). We could not observe a significant difference in CTC numbers detected (*P* = 0.161). In 54.5% of the samples, more CTCs were detected with EpCAM-based enrichment and in 45.5% we found more CTCs using the Trop-2 and CD-49f-based enrichment (Fig. 4b).

Subsequently, we analysed the prognostic relevance of CTCs enriched with the two workflows and applied a cut-off of 5 CTCs per 7.5 ml blood. For CTCs enriched EpCAM based, patients with ≥5 CTCs per 7.5 ml blood had significantly lower PFS (*P* = 0.039) and OS (*P* < 0.001) (Fig. 4c, d). Similarly, patients from whom 5 or more CTCs per 7.5 ml blood were detected upon the EpCAM-independent enrichment also had significantly lower PFS (*P* = 0.047) and OS (*P* = 0.049) (Fig. 4e, f). Interestingly, applying a cut-off of 1 CTC did not lead to significant differences in PFS and OS for both, either the EpCAM or the Trop-2/CD-49f-based enrichment (Supplemental Fig. 8).

Next, we compared the morphology of the EpCAM dependently and independently enriched CTCs and their chromosomal aberrations and mutations. To investigate morphological differences the images taken by the CellSearch CellTracks device were compared. We observed a significantly smaller tumour cell size (*P* = 0.007) (Fig. 4g and Supplemental Fig. 9A) and a significantly lower tumour cell circularity (*P* = 0.002) (Fig. 4h and Supplemental Fig. 9B) of Trop-2/CD-49f-based enriched CTCs. Importantly, processing tumour cells with the two workflows did not affect the tumour cell size or tumour cell circularity differently, as assessed on spiked cell line cells (Supplemental Fig. 10). No significant differences were found regarding the mean cytokeratin staining intensity (*P* = 0.231) (Fig. 4i and Supplemental Fig. 9C).

To investigate the clonal relationship of EpCAM dependently and independently enriched CTCs, low- pass sequencing from one to five CTCs of each group from four patients (patients’ characteristics are listed in Supplemental Table 5) was performed. Upon applying hierarchical clustering, EpCAM dependently and independently enriched CTCs did not separate. However, in patient 8 one EpCAM independently enriched CTC did not cluster to the EpCAM-based enriched CTCs (Fig. 4j). Next mutational hotspot regions were investigated in CTCs from eight patients. Mutations were detected in CTCs from six patients. Three of them appeared in more than one CTC (patient 2 *PIK3CA* H1047L, patient 5 *ERBB2* V777L and patient 6 *ESR1* N532D). Two of them, *PIK3CA* H1047L and *ERBB2* V777L, are well-known mutations, which often occur in breast cancers and lead to the activation of the respective protein. The *ESR1* N532D mutation has not yet been described in breast cancer. By showing its absence in germline DNA, we confirmed its tumoric identity. In all three cases, the respective mutation was present in both EpCAM dependently and independently enriched CTCs. Therefore, no differences of the two CTCs groups regarding the occurrence of mutations were detected (Fig. 4k and Supplemental Fig. 11).

Altogether, these observations lead to the conclusion that EpCAM dependently and independently enriched CTCs originate from identical tumour cell clones that most likely represent a tumour surrogate material of similar relevance. However, we observed patient samples, from which no CTCs were detected using an EpCAM-based enrichment. Therefore, an EpCAM-independent enrichment may close the gap to detect predictive information in such cases.

### Targeting Trop-2 and CD-49f captures additional CTCs that are not detected by an EpCAM-based enrichment

The result shown above can be either explained by an additional value of CTCs enriched EpCAM independently or may only indicate the advantage of enriching CTCs from higher blood volumes as more material was analysed if two methods are performed in parallel. To confirm the former, we established a sequential analysis of at first an EpCAM-dependent and secondly the EpCAM-independent approach targeting Trop-2 and CD-49f. Similar approaches to enrich CTCs from the EpCAM-depleted sample fraction (EDF) were previously already performed by us and others [15, 17, 21].

**Fig. 5:**
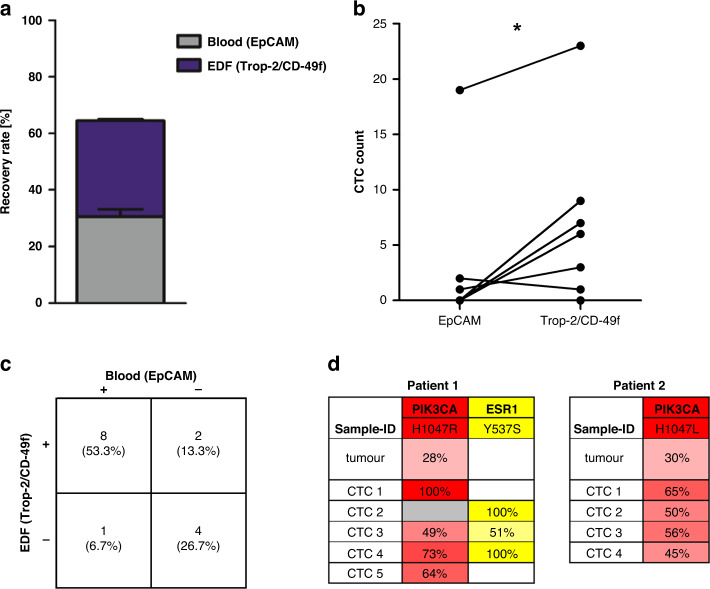
Analysis of CTCs of the EpCAM-depleted fraction from the CellSearch system. **a** Cells from MDA-MB-231 cell line were spiked into healthy donors’ blood and first enriched EpCAM-based. The EpCAM-depleted fraction (EDF) was collected and the remaining tumour cells were subsequently enriched based on Trop-2/CD-49f. **b** EpCAM-depleted fractions from seven patients were collected and divided into two parts. From one part, CTCs were enriched EpCAM-based and from the other part, CTCs were enriched based on Trop-2/CD-49f. The *P* value was determined by paired two-sided *t* test (* indicates a *P* value <0.05). **c** CTC positivity rates of EpCAM-based enrichment from blood and subsequent Trop-2/CD-49f-based enrichment from the EpCAM-depleted fraction of 15 metastatic breast cancer patients. **d** Mutations detected by targeted next-generation sequencing on DNA from CTCs from the EpCAM-depleted fraction and primary tumour tissue. Values indicate the variant allele frequency. Cutoffs of 12.5% or 5% were applied for CTCs or tissue samples, respectively. The grey box indicates that the respective region was not covered in that sample.

To assess the capturing rate of such an approach, tumour cells from the EpCAM low-expressing cell line MDA-MB-231 were spiked into healthy donors’ blood and first enriched EpCAM dependently with the CellSearch system: a recovery rate of 30.6% was obtained. Subsequently, tumour cells from the EpCAM-depleted fraction were again enriched with the CellSearch system, but this time EpCAM independently targeting Trop-2 and CD-49f. This increased the recovery rate by another 33.9% to a total of 64.5% (Fig. 5a).

Next, EDFs were collected from seven samples from metastatic breast cancer patients and tumour cells therefrom were in parallel enriched both EpCAM dependently and independently. Using the Trop-2/CD-49f-based enrichment we detected significantly more CTCs compared to a second enrichment targeting EpCAM (*P* = 0.034) (Fig. 5b).

Subsequently, we analysed EDFs from 15 metastatic breast cancer patients (patients’ characteristics are listed in Supplemental Table 6). We found CTCs in EDF samples from 10 patients (66.7%) and detected up to 8260 CTCs in a 7.5 ml blood equivalent. Moreover, in two of those samples, no CTCs were detected with the prior EpCAM-based enrichment on the CellSearch system (Fig. 5c).

Further, we performed a sequencing analysis on CTCs isolated from the EDF of two patients whose primary tumour displayed a mutation in the *PIK3CA* gene—either H1047R or H1047L—with a variant allele frequency of 28% or 30%, respectively. In both cases, the respective mutation was also found in all analysed CTCs if the fragment was covered: the CTCs harboured the mutations with variant allele frequencies of 49–100%. Moreover, the activating *ESR1* mutation Y537S was detected in three out of five CTCs from patient 1 (Fig. 5d). This mutation was undetectable in the primary tumour and was most likely induced by an oestrogen deprivation therapy that the patient received prior to CTC sampling, indicating acquired resistance to endocrine therapy (Supplemental Fig. 12).

Consequently, these results show that Trop-2/CD-49f-based enriched CTC have an additional clinical value, since this EpCAM-independent enrichment targets CTCs that are not captured by EpCAM-based methods and, moreover, may harbour clinically relevant information, such as predictive mutations.

## Discussion

Using CTCs as a prognostic and predictive factor to optimise breast cancer treatment is an appealing concept. EpCAM is a transmembrane glycoprotein that mediates homotypic cell contact in epithelial tissues and is involved in cell proliferation, stemness and EMT [22]. EpCAM is highly expressed in most epithelial cells including most carcinomas while being absent in blood cells. Therefore, antibodies targeting EpCAM have been established many years ago to capture CTCs and proven to capture tumour cells from the blood whose numbers have a high prognostic value [7, 23]. However, even in the metastatic situation no or only few CTCs are detected in many patients which is at least partly due to the CTCs downregulation or loss of the EpCAM expression [24]. Consequently, many patients cannot benefit from a CTC analysis to date, for example in terms of detecting mutations that indicate therapy resistance or possible treatment targets. Either analysing larger blood volumes—an approach addressed by the diagnostic leukapheresis [25, 26]—or capturing additional CTC populations can overcome this challenge. The analysis of further EpCAM-negative or low-expressing CTCs may offer to close that gap. Here we established a workflow relying on Trop-2 and CD-49f as targets for specific antibody-based capture of CTCs. The resulting EpCAM low and high-expressing CTCs were characterised and EpCAM dependently and independently enriched CTCs were compared regarding their prognostic and predictive values.

Trop-2 is a calcium signal transducer overexpressed in many cancers. A high Trop-2 expression is associated with a worse prognosis and an increased risk for developing metastasis in breast cancer and other cancer subtypes [27]. Moreover, Trop-2 is considered as a therapeutic target and a drug antibody conjugate targeting Trop-2— sacituzumab govitecan—has recently been approved to treat metastatic triple-negative breast cancer [28]. The expression of Trop-2 correlates positively with E-Cadherin and negatively with mesenchymal markers [29]. CD-49f, also known as Integrin α6, is upregulated in many cancer types and promotes migration and invasion. The overexpression of ITGA6 is associated with a worse prognosis [30]. CD-49f is an identifier of cancer stem cells in various tissues and plays an important role in sustaining the self-renewal of cancer stem cells by interconnecting them with the tumorigenic microenvironment [31]. An antibody targeting CD-49f has been applied in combination with the detection of CK8, CK18 and CK19 to identify breast cancer cells of a mesenchymal phenotype in the blood [32].

We have observed a synergistic effect when applying the combination of both antibodies to target Trop-2 and CD-49f. This indicates that both proteins act as complementary enrichment targets facilitating the capture of epithelial-like and mesenchymal-like CTCs. We hypothesise that the synergistic effect results from the capture of hybrid state CTCs that express neither Trop-2 nor CD-49f sufficiently high to be enriched by one of the antibodies alone. These CTCs might only be loaded with a sufficient amount of magnetic particles if both antibodies are used. In cell lines, this effect was not observed, which may be because most cell lines—including those used in our experiments—are very homogenously expressing Trop-2 and CD-49f. Therefore, those cells are captured either by targeting Trop-2 or by targeting CD-49f.

To further increase the validity and reproducibility of our EpCAM-independent CTC enrichment approach, we transferred it to the CellSearch system enabling a partly automated enrichment combined with an automated staining and software-supported identification of CTCs, which will increase the utility in a clinical setting.

Many studies have shown that CTCs enriched with the CellSearch system based on their EpCAM expression have a high prognostic value in metastatic as well as early breast cancer [2, 3]. Interestingly, although only a small patient cohort was analysed, our data indicate that particularly the presence of EpCAM high-expressing CTCs is associated with a poor overall and progression-free survival. The presence of EpCAM low-expressing CTCs was however not associated with the outcome of the patients. These observations are in line with results published by the CTC-Trap programme: In this study, CTCs were enriched from the EDF of prostate and breast cancer patients collected from the CellSearch system. For 32% of the breast cancer patients more than 5 CTCs were identified in the EDF, but no correlation with the overall survival was observed [17]. Of note, in this and other previous studies on the capture of CTCs from the CellSearch system’s EDF it was not demonstrated that the EpCAM-independent enrichment leads to an increased CTC yield compared to a second EpCAM-based enrichment step to elucidate the number of EpCAM-positive CTCs not captured by the initial CellSearch approach. By splitting the EpCAM-depleted fraction, we could show that indeed a fraction of CTCs not enriched with EpCAM-directed antibodies could be captured.

We observed that CTCs retrieved EpCAM independently were smaller and less circular. Such morphological changes could impede the enrichment of mesenchymal-like CTCs by a filtration-based platform. Therefore, this observation indicates an advantage of surface marker-based enrichment strategies for the enrichment of EpCAM low-expressing CTCs.

EpCAM low-expressing CTCs may play a particular role in the development of brain metastasis in breast cancer patients. It was shown that EpCAM-negative CTCs not captured by the CellSearch system harbour a specific protein signature that is suggestive of their metastatic competency to the brain [33]. Moreover, this CTC population includes a stem cell fraction characterised by cancer dormancy markers that may be used to identify patients who are at high risk of developing brain metastasis [34]. In this context, using the cancer stem cell-related protein CD-49f as an enrichment marker may be particularly of interest to capture tumour cells characterised by stem cell-like properties that can act as metastasis-initiating cancer cells or may be cultured in vitro. Of note and unlike CD-49f, the expression of the breast cancer stem cell marker CD44 was observed to be increased in the fraction of EpCAM-positive CTCs. To evaluate the potential of EpCAM-negative CTC as a prognostic and predictive biomarker in early breast cancer further studies need to be performed.

Since here we investigated CTCs from metastatic breast cancer patients, the focus of the study was not on the CTCs’ ability to initiate metastasis. At this stage of the disease, CTCs rather are of particular interest as a tumour surrogate material representing tumour heterogeneity and tumour evolution. We observed a close clonal relationship of EpCAM high-expressing and low-expressing CTCs, as well as EpCAM dependently and independently enriched CTCs on the level of chromosomal aberration, indicating that both fractions of CTCs represent similar subclones of the tumour lesions. In line, we detected widely identical mutations in the EpCAM low and high-expressing CTCs as well as in the EpCAM dependently and independently enriched CTCs which implies that the EpCAM expression may be regulated differently in CTCs representing similar genomic clones by for example epigenetic alterations such as DNA methylation or histone modifications [35].

Despite this observation, previous studies showed that the mutational status of CTCs from the EpCAM-depleted fraction of the CellSearch system can also differ from that of EpCAM-based enriched CTCs [21]. This may be particularly relevant if only few CTCs are analysed or only a minor subclone carries a certain mutation. In such cases, increasing the number of analysed CTCs by the analysis of all subpopulations of CTCs will enhance the probability to detect mutations that are relevant for treatment. The analysis of EpCAM independently captured CTCs is especially pertinent if no CTCs are detected relying on EpCAM-based technologies. Since EpCAM high-expressing and low-expressing CTCs display similar genomic tumour cell clones, we hypothesise that both CTC subgroups will be of equal predictive value regarding genomic parameters. This hypothesis has to be tested in future clinical studies.

In conclusion, our data indicate that EpCAM low-expressing CTCs are less prognostically relevant compared to their EpCAM high-expressing counterparts. However, since they mainly represent similar genomic clones of the tumour as EpCAM high-expressing CTCs, they could equally act as a tumour surrogate material and could especially be of relevant value if no CTCs are detected with EpCAM-dependent technologies. The number of patients benefitting from a CTC-based liquid biopsy can be enlarged by applying antibodies targeting two complementary surface proteins. Moreover, we here present a workflow that enables to include other antibodies than EpCAM to the CellSearch-based CTC enrichment. Thus, this study can act as a proof of concept to target further surface proteins on breast cancer and other cancers’ CTCs by making use of the benefits of an automated CTC enrichment and detection system.

## Materials and methods

### Patients

Metastatic breast cancer patients were enrolled into the Augusta study (approved by the Ethics Committee of the Medical Faculty of the Heinrich Heine University Düsseldorf; Ref-No.: 3430), the DETECT-III study (NCT01619111), the DETECT-IV study (NCT02035813) or the DETECT-V study (NCT02344472).

All patients gave their written informed consent to use their blood samples for CTC analysis and translational research projects. Patients’ characteristics were anonymized with sample identifiers.

### Database analysis

Single CTC RNA sequencing data were obtained from gene expression omnibus (national centre for biotechnology information; GSE144494 and GSE144495). Read counts were normalised to transcripts per million. The expression data were analysed for deviations from a normal distribution using the Shapiro–Wilk test. As normal distributions of expression data were rejected based on *P* values <0.05, non-parametric Spearman correlation coefficients were calculated for the expression of genes of interest in both datasets. Further, the gene expressions of the chosen markers were sorted to an EPCAM-positive and negative fraction, respectively. An *EPCAM* expression of zero was defined as EPCAM-negative and expression levels >0 were defined as *EPCAM* positive. A non-parametric, two-sided Mann–Whitney test was performed. Marker expression levels were compared between the EPCAM-positive and EPCAM-negative fraction using the non-parametric, two-sided Mann–Whitney test; the Benjamini–Hochberg correction was applied to counteract alpha error accumulation due to multiple testing.

### Cell lines

Breast cancer cells from cell lines MDA-MB-231, T-47D, MCF7 and SK-BR-3 were used (ATCC, Manassas, USA; catalogue numbers: MDA-MB 231: HTB-26, SK-BR-3: HTB-30, T-47D: HTB133, and MCF7: HTB-22) and cultured in RPMI 1640 containing 10% foetal calf serum and 1% penicillin–streptomycin (all Thermo Fisher Scientific, Waltham, USA). Cells were grown at 37 °C in a humidified atmosphere with 5% CO_2_ and were detached from the culture flask with an enzyme-free cell dissociation buffer (Thermo Fisher Scientific). Cell lines were authenticated via short tandem repeat analysis and cultured cells were regularly tested negative for *Mycoplasma* infection.

### Flow cytometry

The expression of Trop-2 and CD-49f on cell lines was assessed with a CyAN ADP flow cytometer (Beckmann Coulter, Brea, USA). Cells were stained with antibodies against Trop-2 (clone 162-46, Cat#: 551317, BD Biosciences, Franklin Lakes, USA; Isotype: Mouse IgG1, Cat#: 555756, BD Biosciences) and CD-49f (clone GoH3, Cat#: ab105669, Abcam, Cambridge, UK; Isotype: Rat IgG2a, Cat#: ab18450, abcam). As secondary antibodies AlexaFluor coupled anti-mouse (anti-mouse IgG, AlexaFluor 488 conjugated, Cat#: A11001, Thermo Fisher Scientific) and anti-rat antibodies (anti-rat IgG, AlexaFluor 546 conjugated, Cat#: A11081, Thermo Fisher Scientific) were used.

### CTC enrichment and identification

EpCAM-dependent enrichment of CTCs was performed using the CellSearch Circulating Epithelial Cell Kit (Menarini, Florence, Italy) according to the manufacturer’s instructions on the Celltracks Autoprep System (Menarini). In total, 7.5 ml of blood were used for enumeration of CTCs. Identification was performed using the Celltracks Analyzer II (Menarini).

For the EpCAM-independent enrichment, first, the mononuclear cells were enriched from 5 ml blood by density-gradient centrifugation using SepMate PBMC Isolation Tubes (Stemcell Technologies, Vancouver, Canada). Next, tumour cells were labelled with biotinylated antibodies against Trop-2 (clone REA916, biotin-conjugated, Cat#: 130-115-054, Miltenyi, Bergisch Gladbach, Germany) and CD-49f (clone REA518, biotin-conjugated, Cat#: 130-123-243, Miltenyi). Afterwards, tumour cells were enriched on either the Isoflux system or the CellSearch system. For Isoflux-based enrichment, cells were incubated with Cellection Biotin Binder-beads (Thermo Fisher Scientific) added to an Isoflux cartridge (Fluxion Biosciences, Alameda, USA) and retrieved on the Isoflux system (Fluxion Biosciences). Upon retrieval, cells were either spun and stained on glass slides or stained in suspension for nucleic acid (DAPI; F. Hoffmann-La Roche, Basel, Switzerland), cytokeratins (clones C11/AE1/AE3, TRITC conjugated, Cat#: CKALLRMB000S, Aczon, Monte San Pietro, Italy), CD45 (clone 35-ZS, AlexaFluor 647 conjugated, Cat#: sc-1178 AF647, Santa Cruz Biotechnology, Dallas, USA), and EpCAM (clone VU1D9, AlexaFluor 488 conjugated, Cat#: 5488S, Cell Signaling Technology). Afterwards, beads were released using DNase (Thermo Fisher Scientific). CTCs were identified on a fluorescence microscope (CKX41, Olympus, Tokyo, Japan). For DNA analysis cells were released from the beads prior to staining to avoid DNA degradation. For the CellSearch-based enrichment, cells coupled with biotinylated antibodies were transferred to CellSearch tubes (Menarini), centrifuged and retrieved on the Celltracks Autoprep system. A modified CellSearch Circulating Epithelial Cell Kit was used: tube 1 was replaced with streptavidin-coupled ferrofluid (BioMagnetic Solutions, State College, USA) and tube 2 was replaced with PBS (Thermo Fisher Scientific). Enumeration of CTCs was performed with the Celltracks analyzer II (Menarini).

For analysis of CTCs from the EpCAM-depleted fraction, this fraction was collected from the Celltracks Autoprep System using the Automated Sample Collection Device [36]. Collected samples were centrifuged and processed similarly to blood.

Spike-in experiments were used to establish the workflows and were regularly performed as controls. Therefore, 500 cells (unless otherwise stated) of the respective cell line were added to blood samples of healthy donors, who were enrolled on the Augusta study and gave their written informed consent for the use of their blood samples for spike-in experiments and to serve as controls in translational research projects.

### Image analysis

Analysis of fluorescence intensities, tumour cell sizes, and cell shapes was performed using ImageJ [37].

### CTC isolation and whole-genome amplification

Single CTCs were isolated from the CellSearch cartridge by micromanipulation with the CellCelector (ALS, Jena, Germany). Cells were deposited in PCR tubes to perform whole-genome amplification (WGA) as described previously [38].

DNA of single isolated cells was amplified by WGA with the Ampli1 WGA Kit (Menarini) according to the manufacturer’s protocol. Afterwards, DNA integrity was determined with the Ampli1 QC Kit (Menarini).

### Low-pass sequencing

Single-cell WGA products of the highest quality were prepared for copy number variation (CNV) analysis by the Ampli1 LowPass kit for Illumina (Menarini) and sequenced on a MiSeq system (Illumina, San Diego, USA) [39]. CNV profiles were generated using an automated inhouse pipeline which contains in brief the following steps: (1) trimming of raw FASTQ files with BBDuk 38.76 [40], (2) read decontamination using BioBloom Tools 2.0.13 [41], (3) mapping with bwa 0.7.17 [42] and (4) CNV profil generation applying QDNAseq 1.24.0 [43]. After each step, quality control was performed.

### Mutation analysis

Predictive mutations in the *PIK3CA*, *ESR1*, *AKT1* and *ERBB2* genes were analysed. Hotspot regions were amplified by a multiplex PCR, barcoded with Multiplicom MID Dx primers (Agilent Technologies, Santa Clara, USA) and sequenced on a MiSeq system as described previously [44].

To confirm the somatic origin of the *ESR1* N532D mutation germline DNA of the patient whose CTCs harboured that mutation was sequenced. The DNA was extracted from WBCs using the QIAamp DNA Mini Kit (Qiagen, Venlo, Netherlands).

### Statistical analysis

Statistical analysis was performed using GraphPad Prism (GraphPad Software, San Diego, USA) or Origin (Origin Lab, Northampton, USA). Information on the statistical tests applied are given in the figure legends. A *P* value <0.05 was considered as statistically significant. The equality of variances was tested by F test.

## Supplementary information

Supplemental Material

## Data Availability

All raw data can be requested from the corresponding author.
