# Supplementary material for: Comparative analysis of EpCAM high-expressing and low-expressing circulating tumour cells with regard to their clonal relationship and clinical value
Source: Br J Cancer. 2023 Feb 23;128(9):1742–52. doi: 10.1038/s41416-023-02179-0 (PMC10133238; doi:10.1038/s41416-023-02179-0)
Supplement: Supplementary file 1 — Supplemental Material [file 41416_2023_2179_MOESM1_ESM.docx]

Supplementary Material

Supplemental Table 1: Clinical characteristics of patients included in the analysis of the EpCAM expression on CTCs at time of diagnosis

na, no information available; NST, no special type

| Patient ID | Age at diagnosis | Tumor size | Nodal status at time of diagnosis | Metastasis status at time of diagnosis | Histology | Grading | Subtype |
| --- | --- | --- | --- | --- | --- | --- | --- |
|  |  |  |  |  |  |  |  |
| 1 | 36 | Tis | N0 | M0 | NST | G3 | HER2 enriched |
| 2 | 75 | T1 | N1 | M1 | Invasive lobular | G2 | Luminal |
| 3 | na | T2 | N0 | M1 | Invasive lobular | G2 | Luminal |
| 4 | 43 | T3 | N3 | M0 | NST | G2 | Triple negative |
| 5 | 68 | T1 | N0 | M0 | NST | na | Luminal |
| 6 | 35 | T1 | N1 | M0 | NST | G2 | Luminal |
| 7 | 49 | T2 | N1 | M0 | NST | G3 | Luminal |
| 8 | 59 | T2 | N1 | M1 | Invasive lobular | G2 | Luminal |
| 9 | 33 | T3 | N2 | M0 | Invasive lobular | G3 | Triple negative |
| 10 | 58 | T2 | N0 | M0 | NST | G2 | Luminal |
| 11 | na | na | na | na | na | na | na |
| 12 | na | na | na | na | na | na | na |
| 13 | 51 | T2 | N0 | M1 | na | na | HER2 enriched |
| 14 | 66 | T2 | N2 | M0 | Invasive lobular | G2 | HER2 enriched |
| 15 | na | T2 | N0 | M0 | NST | G3 | Triple negative |
| 16 | 45 | na | na | M1 | na | na | HER2 enriched |
| 17 | 74 | na | na | M1 | na | na | Luminal |
| 18 | na | na | na | na | na | na | na |
| 19 | na | na | na | na | na | na | na |
| 20 | na | na | na | na | na | na | na |
|  |  |  |  |  |  |  |  |

Supplemental Table 2: Analysis of association of detection of EpCAM high or EpCAM low expressing CTCs with clinical data

The p-values were determined by Chi-square test and Fisher's exact test.

na, no information available; NST, no special type

|  |  | EpCAM high CTCs, n (%) |  | EpCAM low CTCs, n (%) |  | overall | na |
| --- | --- | --- | --- | --- | --- | --- | --- |
|  |  |  |  |  |  |  |  |
| Histology | NST | 1 (14.3) |  | 3 (42.9) |  | 7 | 8 |
|  | invasive lobular | 3 (60.0) | *p* = 0.0977 | 3 (60.0) | *p* = 0.5582 | 5 |  |
|  |  |  |  |  |  |  |  |
| Hormone receptor | positive | 3 (27.3) |  | 6 (54.5) |  | 11 | 5 |
|  | negative | 2 (50.0) | *p* = 0.4090 | 1 (25.0) | *p* = 0.3104 | 4 |  |
|  |  |  |  |  |  |  |  |
| HER2/neu | positive | 0 (0.0) |  | 1 (25.0) |  | 4 | 5 |
|  | negative | 5 (45.5) | *p* = 0.2308 | 6 (54.5) | *p* = 0.3104 | 11 |  |
|  |  |  |  |  |  |  |  |
| Metastasis location | bones or bones + visceral | 3 (75.0) |  | 2 (50.0) |  | 4 | 10 |
|  | visceral only | 1 (16.7) | *p* = 0.0651 | 2 (33.3) | *p* = 0.5982 | 6 |  |
|  |  |  |  |  |  |  |  |

Supplemental Table 3: Clinical characteristics of patients included in the DNA analysis of EpCAM high and low expressing CTCs

na, no information available; NST, no special type

| Patient ID | Age | UICC at time of diagnosis | histology | Grading | Subtype |
| --- | --- | --- | --- | --- | --- |
|  |  |  |  |  |  |
| 1 | 77 | II | invasive-lobular | G2 | Luminal |
| 2 | 72 | III | NST | G2 | Luminal |
| 3 | 46 | III | na | na | Luminal |
|  |  |  |  |  |  |

Supplemental Table 4: Clinical characteristics of patients included in the comparison of EpCAM dependently and independently enriched CTCs at time of diagnosis

na, no information available; NST, no special type

| Characteristics |  | Total | in % |
| --- | --- | --- | --- |
|  |  |  |  |
| Patients |  | 22 | 100 |
|  |  |  |  |
| Age |  |  |  |
| Mean | 54.4 |  |  |
| Median | 56.5 |  |  |
| Range | 36 - 72 |  |  |
|  |  |  |  |
| Tumor size |  |  |  |
| T1 |  | 3 | 13.6 |
| T2 |  | 11 | 50.0 |
| T3 |  | 4 | 18.2 |
| T4 |  | 3 | 13.6 |
| na |  | 1 | 4.5 |
|  |  |  |  |
| Nodal status |  |  |  |
| 0 |  | 10 | 45.5 |
| 1 |  | 1 | 4.5 |
| 2 |  | 4 | 18.2 |
| 3 |  | 3 | 13.6 |
| na |  | 4 | 18.2 |
|  |  |  |  |
| Metastasis status |  |  |  |
| 0 |  | 13 | 59.1 |
| 1 |  | 8 | 36.4 |
| na |  | 1 | 4.5 |
|  |  |  |  |
| Histology |  |  |  |
| NST |  | 4 | 18.2 |
| Invasive lobular |  | 7 | 31.8 |
| Invasive mucinous |  | 1 | 4.5 |
| na |  | 10 | 45.5 |
|  |  |  |  |
| Grading |  |  |  |
| 2 |  | 12 | 54.5 |
| 3 |  | 3 | 13.6 |
| na |  | 7 | 31.8 |
|  |  |  |  |
| Subtype |  |  |  |
| Luminal |  | 15 | 68.2 |
| Her2 Enriched |  | 3 | 13.6 |
| Triple Negative |  | 2 | 9.1 |
| na |  | 2 | 9.1 |
|  |  |  |  |

Supplemental Table 5: Clinical characteristics of patients included in the DNA analysis of EpCAM dependently and independently enriched CTCs

na, no information available; NST, no special type

| Patient ID | Age | UICC at time of diagnosis | Histology | Grading | Subtype |
| --- | --- | --- | --- | --- | --- |
|  |  |  |  |  |  |
| 1 | 55 | IV | NST | G2 | Luminal |
| 2 | 73 | III | NST | G2 | Luminal |
| 3 | 59 | na | na | na | Luminal |
| 4 | 54 | III | NST | 2 | Luminal |
| 5 | 73 | IV | na | 2 | Luminal |
| 6 | 69 | II | na | 3 | Triple negative |
| 7 | 62 | II | Invasive lobular | 3 | Luminal |
| 8 | 53 | II | NST | 3 | Luminal |
|  |  |  |  |  |  |

Supplemental Table 6: Clinical characteristics of patients included in the analysis CTCs from the EpCAM depleted sample fraction at time of diagnosis

na, no information available; NST, no special type

| Characteristics |  | Total | in % |
| --- | --- | --- | --- |
|  |  |  |  |
| Patients |  | 15 | 100 |
|  |  |  |  |
| Age |  |  |  |
| Mean | 49.8 |  |  |
| Median | 45.5 |  |  |
| Range | 32 - 72 |  |  |
|  |  |  |  |
| Tumor size |  |  |  |
| T1 |  | 2 | 13.3 |
| T2 |  | 5 | 33.3 |
| T3 |  | 3 | 20.0 |
| T4 |  | 2 | 13.3 |
| na |  | 3 | 20.0 |
|  |  |  |  |
| Nodal status |  |  |  |
| 0 |  | 2 | 13.3 |
| 1 |  | 3 | 20.0 |
| 2 |  | 3 | 20.0 |
| 3 |  | 2 | 13.3 |
| na |  | 5 | 33.3 |
|  |  |  |  |
| Metastasis status |  |  |  |
| 0 |  | 8 | 53.3 |
| 1 |  | 4 | 26.7 |
| na |  | 3 | 20.0 |
|  |  |  |  |
| Histology |  |  |  |
| NST |  | 1 | 6.7 |
| Invasive lobular |  | 1 | 6.7 |
| na |  | 13 | 86.7 |
|  |  |  |  |
| Grading |  |  |  |
| 2 |  | 6 | 40.0 |
| 3 |  | 2 | 13.3 |
| na |  | 7 | 46.7 |
|  |  |  |  |
| Subtype |  |  |  |
| Luminal |  | 9 | 60.0 |
| Her2 Enriched |  | 2 | 13.3 |
| Triple Negative |  | 1 | 6.7 |
| na |  | 3 | 20.0 |
|  |  |  |  |

Supplemental Figure 1: Evaluation of Trop-2 and CD-49f expression on cell lines

Binding of antibodies targeting Trop-2 and CD-49f to cells from different breast cancer cell lines and white blood cells (WBCs) was determined by flow cytometry. Mean fluorescence intensities for T-47D or MDA-MB-231, respectively, were set as 100%.

Supplemental Figure 2: Tumor cell loss during staining and bead release procedure

Cells from SK-BR-3 cell line were spiked into healthy donors’ blood samples and enriched based on Trop-2 and CD-49f on the Isoflux system. Afterwards cell were first stained and then released from beads. Recovery rates were determined after enrichment, after enrichment and staining, and after enrichment, staining, and bead release. Error bars indicate standard deviation.

Supplemental Figure 3: EpCAM expression of CTCs captured with Antibodies targeting Trop-2 or CD-49f

The *p*-value (*p* = 0.045) was determined by two-way ANOVA.


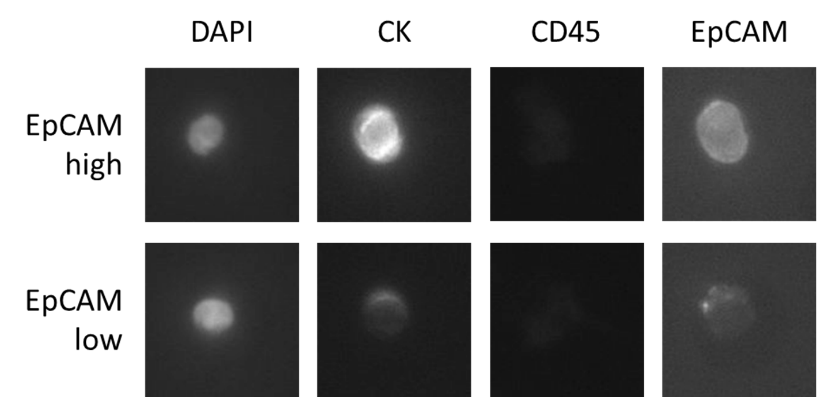


Supplemental Figure 4: Representative images of an EpCAM high expressing and EpCAM low expressing CTCs.

An original magnification of 40× was used.

CK, cytokeratins


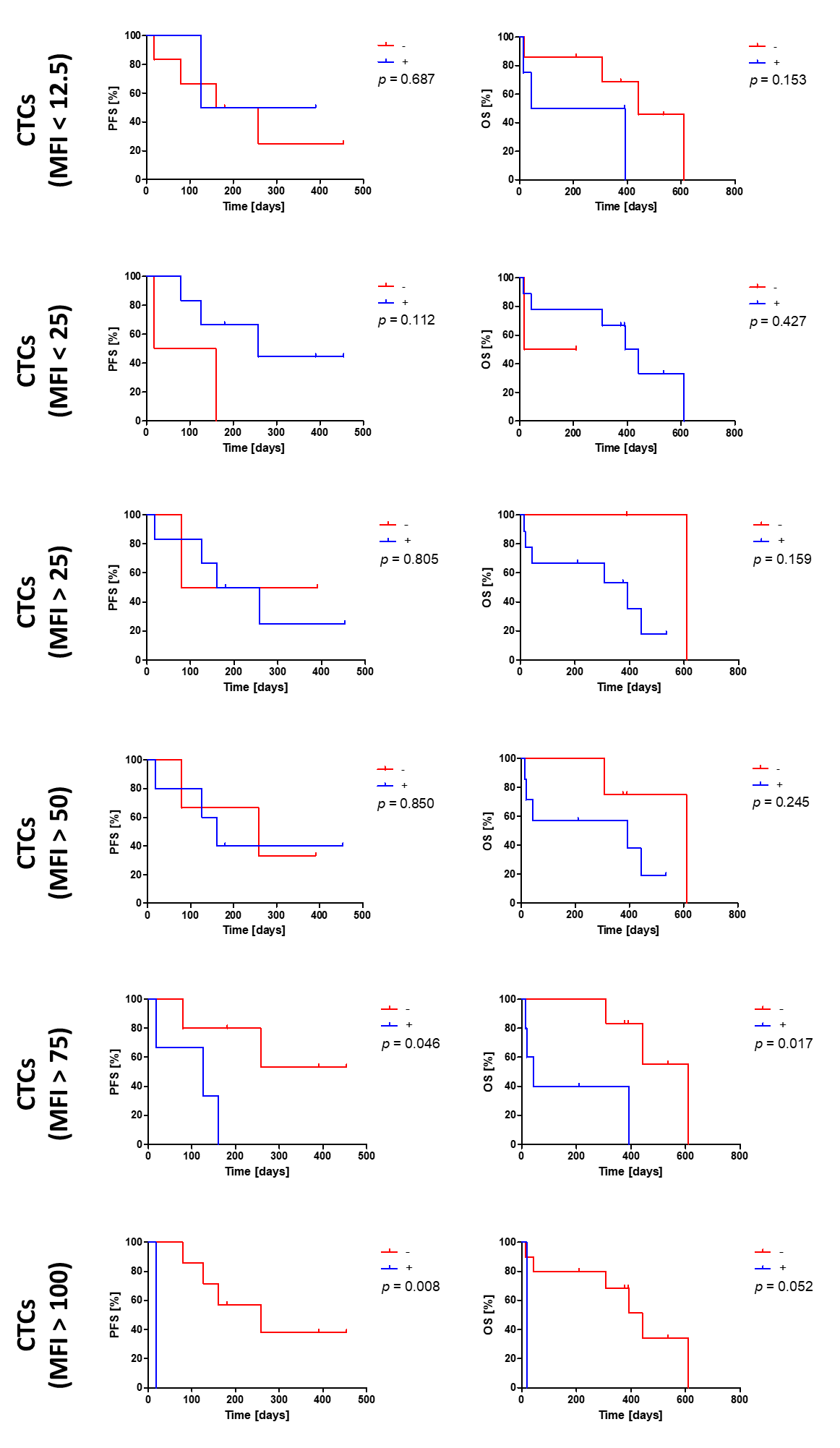


Supplemental Figure 5: Survival analysis by applying different EpCAM fluorescence intensity cutoffs for EpCAM high and low expressing CTCs

Kaplan-Meier-plots of patients with at least one CTC falling below (A & B) or exceeding (C – F) the respective cutoff of the EpCAM mean fluorescence intensities (MFIs). Blue line (+) represents patients in whose samples at least one CTC below the cutoff (row 1 and 2) or above the cutoff (row 3 to 6) was detected. The *p*-values were determined by Log-rank test.

PFS, progression free survival; OS, overall survival


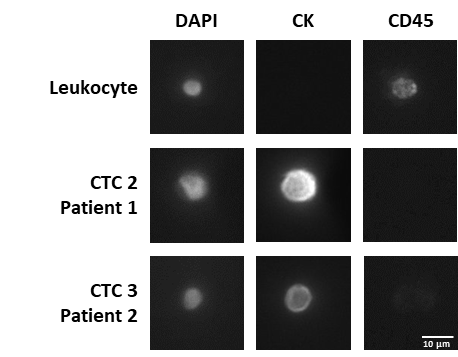


Supplemental Figure 6: Isolated CTC without or with hardly any genomic aberrations

An original magnification of 40× was used.

CK, cytokeratins

Supplemental Figure 7: Recovery rates of spiked cells from SK-BR-3 cell line

10, 100, or 500 cells from SK-BR-3 cell line were spiked into healthy donors’ blood samples and enriched based on Trop-2 and CD-49f on the CellSearch system. Error bars indicate standard deviation. Values indicate recovery rates.


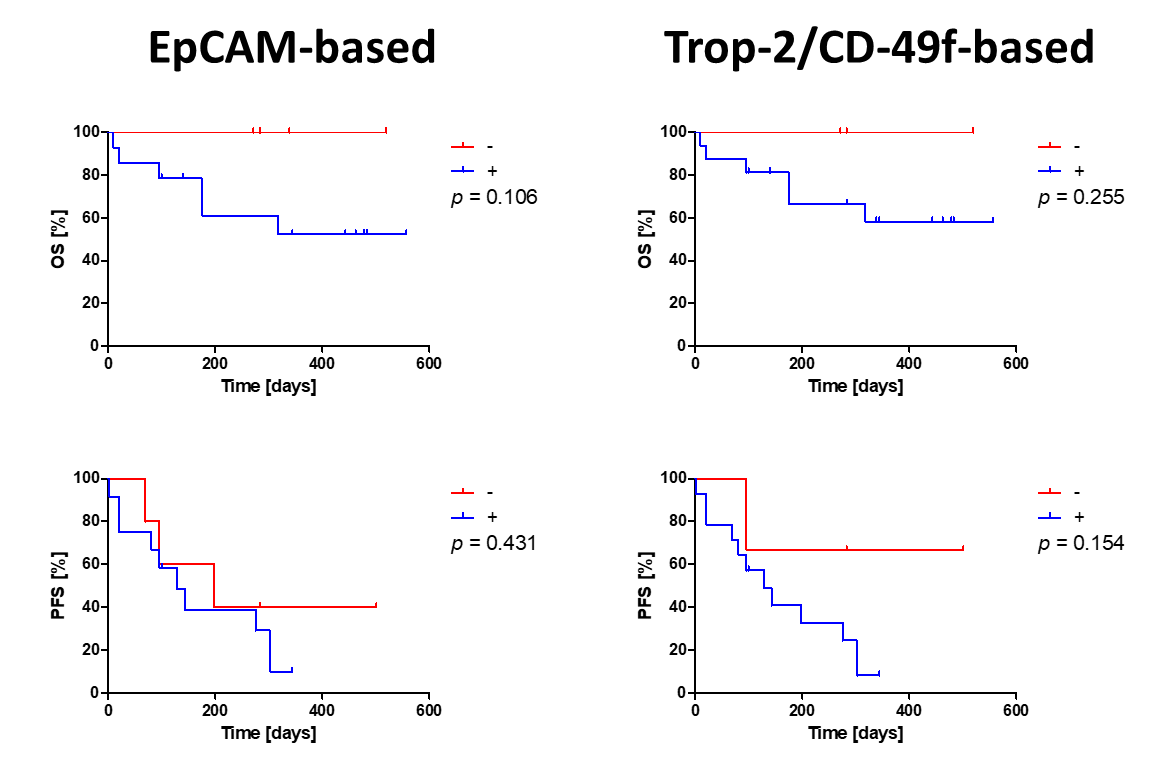


Supplemental Figure 8: Survival analysis by applying a cutoff of 1 EpCAM- or Trop2/CD49f-based enriched CTCs

Kaplan-Meier plots of progression free survival [A] and overall survival [B] of patients with ≥1 CTC versus patients without per 7.5 ml blood based on the EpCAM-based enrichment. Kaplan-Meier plots of progression free [C] and overall survival [D] of patients with ≥1 CTCs versus patients without CTCs in 5 ml blood based on the Trop-2/CD-49f-based enrichment. The *p*-values were determined by Log-rank test.

PFS, progression free survival; OS, overall survival


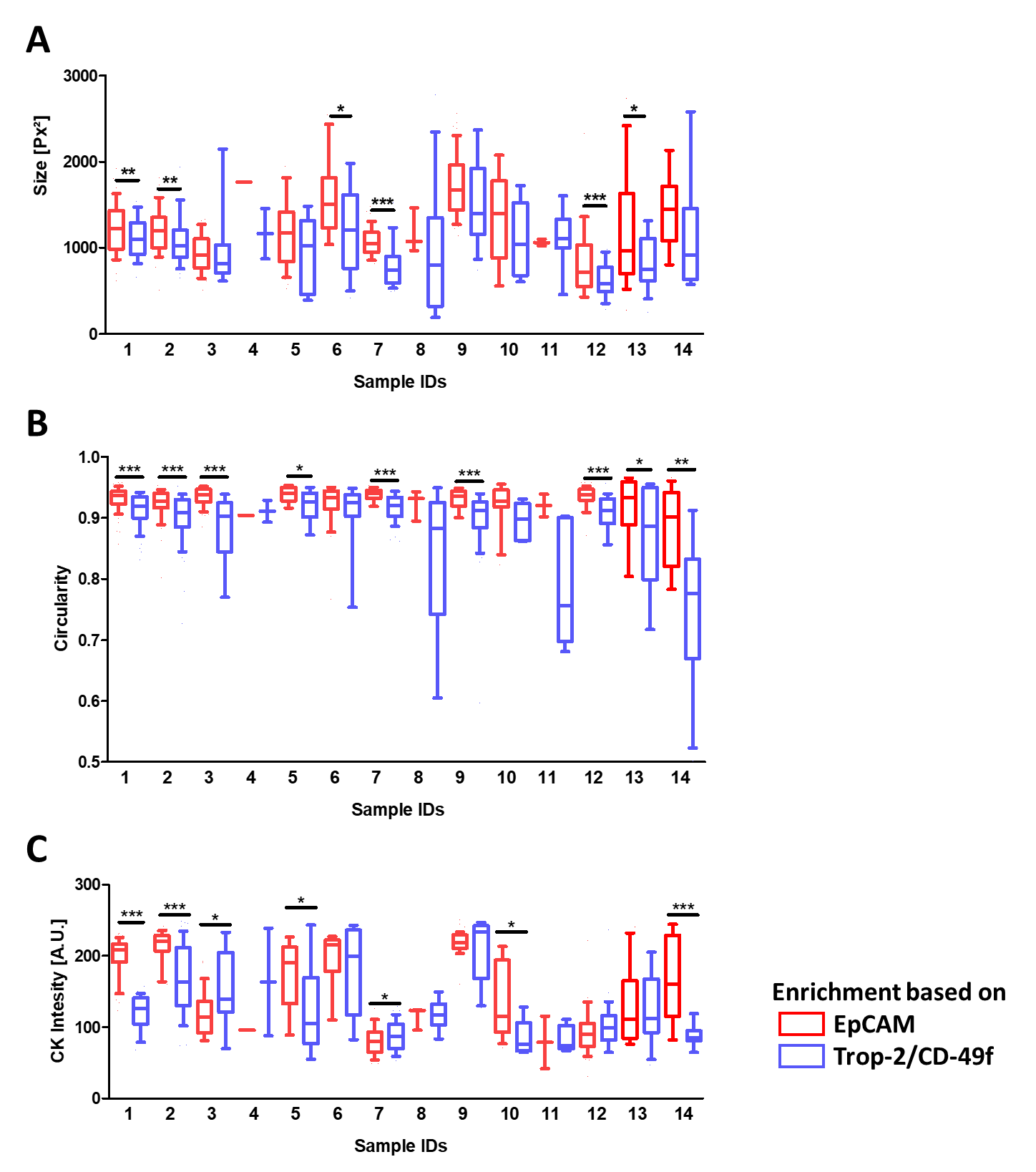


Supplemental Figure 9: Comparison of CTC morphology for each patient

The size [A], the circularity [B] and the cytokeratin fluorescence intensity [C] of CTCs enriched EpCAM-dependently and -independently were compared by two tailed *t*-test (* indicates a *p*-value < 0.05; ** indicates a *p*-value < 0.01; *** indicates a *p*-value < 0.001).


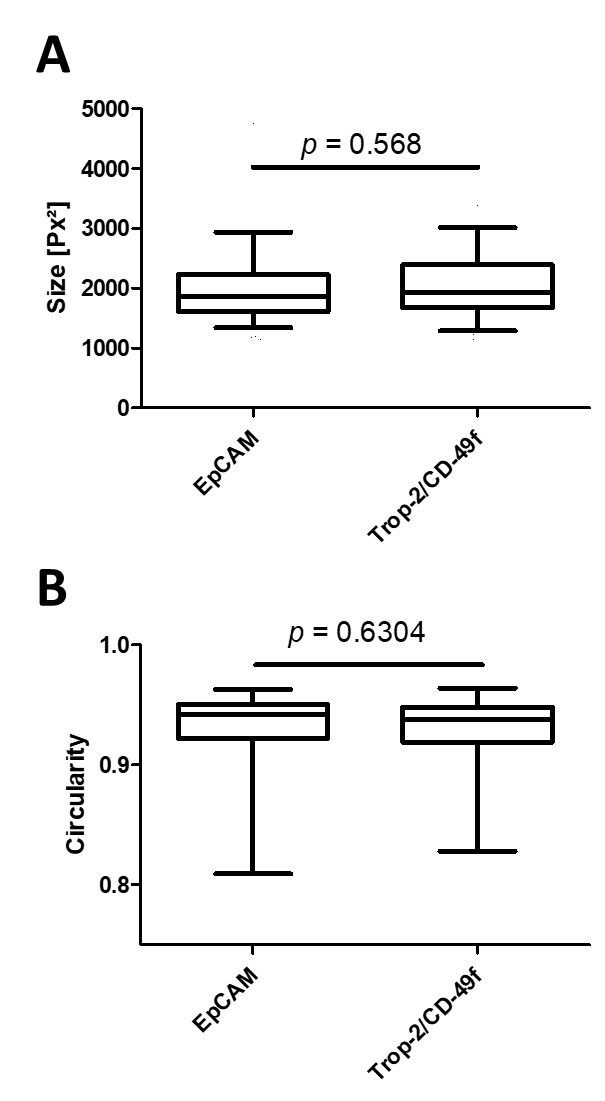


Supplemental Figure 10: Comparison of morphology of EpCAM dependently and independently enriched cells from MDA-MB-231 cell line

The size and circularity of cells from MDA-MB-231 cells spiked into blood and enriched with antibodies targeting EpCAM or Trop2 and CD-49f were compared by two tailed *t*-test.

Supplemental Figure 11: Mutation Analysis of EpCAM- and Trop-2/CD-49f-based enriched CTCs

Mutations detected by targeted NGS sequencing on DNA from EpCAM dependently and independently enriched CTCs. Values indicate the variant allele frequency. A cutoff of 12.5% was applied. Grey boxes indicate that the respective region was not covered in that sample.


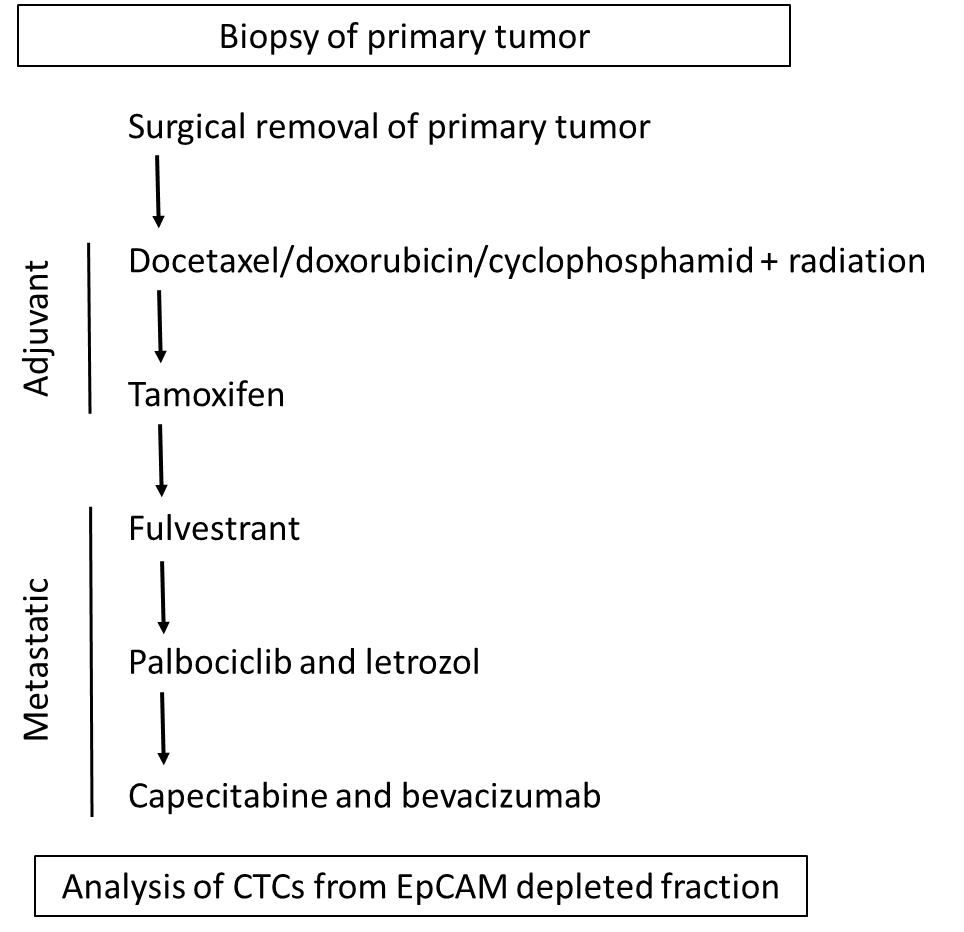


Supplemental Figure 12: Treatment scheme of patient 2 from Figure 5D
